# Transgenerational Epigenetic Inheritance of Early-Life Stress from Grand-Dams Through Paternal Gametes: Impaired Social Cognition and Reduced Reactivity to Aversive Predictors in DAT-HET Rats

**DOI:** 10.3390/biology14091229

**Published:** 2025-09-09

**Authors:** Eleonora D’Antonio, Gioia Zanfino, Concetto Puzzo, Micaela Capobianco, Francesco Mannella, Vincenzo De Laurenzi, Giuseppe Curcio, Walter Adriani

**Affiliations:** 1 Department of Biotechnological and Applied Clinical Sciences, University of L’Aquila, 67100 L’Aquila, Italy; eleonora.dantonio@gmail.com (E.D.); giuseppe.curcio@univaq.it (G.C.); 2 Center for Behavioural Sciences and Mental Health, Istituto Superiore di Sanità (ISS), 00161 Rome, Italy; zanfinogioia@gmail.com (G.Z.); concetto.puzzo@alice.it (C.P.); 3 Department of Economic, Psychological and Communication Sciences, Niccolò Cusano University, 00166 Rome, Italy; micaela.capobianco@unicusano.it; 4 European Mind and Metabolism Association, 00161 Rome, Italy; 5 Faculty of Psychology, International Telematic University Uninettuno (UTIU), 00186 Rome, Italy; 6 Institute of Cognitive Sciences and Technologies (ISTC), Consiglio Nazionale delle Ricerche (CNR), 00185 Rome, Italy; francesco.mannella@istc.cnr.it; 7 Department of Innovative Technologies in Medicine & Dentistry, University of Chieti-Pescara, 66100 Chieti, Italy; delaurenzi@unich.it

**Keywords:** transgenerational inheritance, epigenetic effects, passive avoidance, social cognition, Dorsal/Ventral/Medial PFC, early-life stress, rats’ behavior

## Abstract

Transgenerational epigenetic inheritance has emerged as a compelling mechanism by which early-life stress can shape behavior in descendants with no direct exposure to trauma. However, whether such heritable modifications affect subtle behavioral phenotypes, like processing of social and emotional stimuli, remains poorly understood. In this study, we investigated the behavioral profile of fourth-generation heterozygous dopamine-transporter (DAT-HET) rats. Compared to control (SX) rats, our experimental group (labelled SIKK) consisted of animals (at G4, F3) born from MIK sires (at G3, F2), who descended from grand-dams (at G2, F1) who were in turn exposed to early-life maltreatment by their own DAT-KO mothers (the great-grand-dams, at G1, F0). To probe inhibitory control and social cognition, we employed the signaled licking / avoidance of punishment (SLAP) task, the elicited preference test (EPT), and the social recognition test (SRT). In the SLAP task, SIKK rats exhibited slower acquisition of passive avoidance, suggesting dampened sensitivity to predictive aversive cues. In the EPT, wild-type focal rats displayed a clear preference for SX over SIKK conspecifics, indicating reduced social appeal of epigenetically altered animals. In the SRT, SX rats successfully discriminated between a novel and a familiar DAT-KO conspecific, while SIKK rats failed to do so, revealing impaired social cognition. Together, these findings indicate that, despite the absence of direct trauma in their infancy, SIKK rats exhibit a distinct behavioral phenotype characterized by increased reactivity to threat and deficits in social preferences and cognition. These alterations reflect inherited dysfunctions in limbic dopaminergic circuits, particularly within PFC. Our study highlights how an ancestor’s adversity can shape adaptive behavior in future generations, providing a powerful model for understanding the biological basis of vulnerability to psychiatric disorders.

## Simple Summary

Stressful experiences in early life, when transmitted epigenetically through the pedigree, can affect how individuals behave, even generations later. In this study, we used a group of DAT +/− rats that had their grandparent exposed to poor maternal care. These rats were compared to others with identical pedigree but whose grandparent did not experience a stressful infancy. We tested their ability to avoid danger, their social preferences, and memory of other rats. The rats with a history of ancestral stress learned less quickly to avoid danger, and less efficiently exploited predictors of safe conditions. However, they showed more difficulty in forming clear social preferences, being somewhat avoided by others; also, they were unable to recognize familiar vs. novel companions. These results suggest that early-life trauma can leave long-lasting biological sequelae across generations. Understanding how these phenotypic patterns are inherited may help to identify early markers of emotional vulnerability and contribute to the prevention of mental health disorders.

## 1. Introduction

Epigenetics has become one of the most promising frontiers in studying the interaction between genetics, environmental factors, and behavior. The term was first introduced by Conrad Waddington in the 1940s to describe the processes that mediate between genotype and phenotype during ontogenesis [1]. Over time, the concept has expanded and is now defined as all regulatory mechanisms capable of modifying gene expression without altering the DNA sequence [2]. Many authors underlined the heritability of such epigenetic markers, shaping the modern view of epigenetics as a dynamic gene regulation system, influenced by environmental cues.

Epigenetic modifications (such as DNA CpG methylation, histone acetylation, post-translational “editing” modifications, and non-coding RNA activity) are now widely recognized as crucial in regulating cellular and behavioral processes. Of particular relevance, when such modifications affect germline cells, they can be transmitted across generations, contributing to complex behavioral phenotypes without any direct change in the DNA sequence [3,4,5]. This transgenerational form of epigenetic inheritance has been mostly studied in animal models, where adverse environmental conditions (such as maternal-care deprivation or early-life stress) can be tightly controlled.

Notably, murine studies using the maternal separation with unpredictable stress (MSUS) model have shown that early postnatal trauma in male mice can induce behavioral alterations—including depression-like symptoms and increased risk taking—that persist across multiple generations, even up to the fourth, in the absence of continued environmental stressors [6]. These effects have been linked to epigenetic alterations in sperm RNA and hypothalamic–pituitary–adrenal (HPA) axis (dys)regulation. Similarly, research in precocial birds, such as chickens, has demonstrated that prenatal maternal stress can lead to fear-related behavioral traits in offspring, which are epigenetically inherited across generations [7].

**Figure 1:**
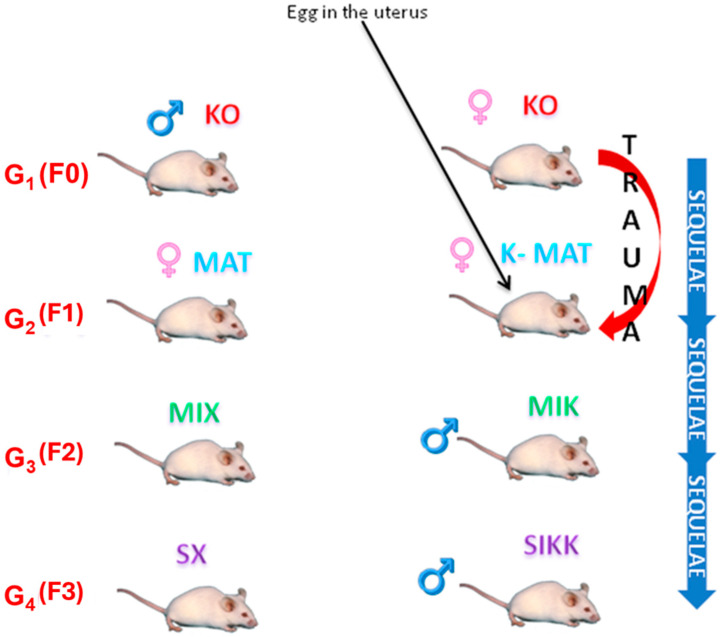
Control rats on the left side (SX rats in the G4, F3) of the diagram and experimental rats on the right side (SIKK rats in the G4, F3). Control lineage has a DAT-KO great-grandfather. The great-grandmother (a DAT-KO hyperactive and obsessive dam at G1, F0) provokes a traumatic insult on G2, F1 (the heterozygous K-MAT grandmother when infant). Within her body develops the uterus where the eggs are located, subsequently generating G3, F2 rats (the heterozygous MIK offspring, later acting as sires). In the G2, F1 and G3, F2 rats, epigenetic sequelae could be conferred directly as a result of the mistreatment (received by the infant G2, F1 pup and her G3, F2 eggs within the uterus). We hypothesize that the putative epimutations confer the sequelae, in turn, to the G4, F3 offspring, i.e., onto SIKK offspring from the MIK sire via his sperm. In summary, to avoid any confounding with maternal care, we used MIK sires (or MIX controls) mated with WT dams for G4, F3 pups. Current study was conducted on fourth-generation SIKK (or their SX control) male rats.

Within this framework, the heterozygous dopamine-transporter (DAT-HET) rat has emerged as a valuable model to explore gene–environment interactions. Compared to wild-type (WT) animals, DAT-HET rats display increased stress sensitivity, lower affiliative behaviors, maternal care deficits, and higher levels of activity. Studies conducted with this model clearly suggest that behavioral alterations may be passed on to the offspring, via epigenetic marks which additionally may differ depending on whether they are transmitted from the sire or from the dam [8,9,10,11]. In previous research by our group [12,13], we demonstrated that specific effects could be transmitted paternally, independent of maternal interaction, particularly when fathers had experienced early-life adversity, being themselves of −/− genotype and/or having been delivered by −/− mothers. Additionally, the latter DAT-KO dams were serendipitously discovered to express dysfunctional caregiving, shown in particular with obsessive–compulsive licking and grooming, with excessive contact from which pups tried to flee away [14]. We proposed this phenomenon to model a “naturalistic” infancy trauma: the adversity being produced spontaneously by the natural DAT −/− dam, without any artificial experimenter intervention. We let these pups become dams and sires, and then grand-dams and grand-sires. This allowed the development of two experimental lines, leading to the current experimental groups: SX (DAT-HET rats, no trauma in the pedigree: controls), and SIKK (presence of trauma in the pedigree, possibly epigenetically “marked” grand-dams and -sires), were now studied in the fourth generation. Throughout the manuscript, we refer to the studied animals as belonging to the “fourth generation” (G4, corresponding to F3) when considering the initial trauma perpetrated by the great-grand-dam (DAT-KO; “first generation” G1 corresponding to F0) upon the grand-dam (k-MAT; “second generation” G2 corresponding to F1), whose male offspring (MIK; “third generation” G3 corresponding to F2) later acted as sires of the current SIKK rats (see Figure 1).

Studying the fourth generation entails that current experimental subjects were not even eggs or sperm at the time of injury. Present rats provide a unique opportunity to assess the epigenetical persistence of non-genetic effects and their influence on cognition and social behavior. We hypothesized that SIKK rats would show altered functioning in limbic brain regions, responsible for integrating external and internal stimuli, particularly the medial prefrontal cortex (mPFC).

The mPFC plays a key role in executive control, emotional regulation, social decision-making, and learning from aversive events [15,16]. This brain region integrates glutamatergic, dopaminergic, and serotonergic inputs from limbic regions and has been linked to top-down control of behavior, including working memory, behavioral flexibility, and avoidance learning. Alterations in mPFC functioning are implicated in psychiatric disorders such as schizophrenia, ADHD, and depression [17,18]. Moreover, pharmacological manipulation of this region has been shown to affect social memory and motivation, as well as coping abilities in response to stress [19,20].

To test these hypotheses, we employed three complementary behavioral paradigms.

In the elicited preference test (EPT), wild-type (WT) focal rats were allowed to freely explore an apparatus with two smaller cages, one empty and one containing a social stimulus, with either a SX or a SIKK epigenetic background. This task measured the ability to discriminate the social attractiveness of a conspecific: this social preference can be modulated in a range between familiar social stimuli and potentially abnormal behavioral signs, coming from distinct individual epigenomes. This task was based on previously validated methods [21].

In the social recognition test (SRT), SX and SIKK focal rats were free to move and exposed to two confined conspecifics (WT and KO) for three days, in order to get familiar with both rats. On the fourth day, the KO stimuli were replaced by a novel KO individual. This setup tested the focal animal’s capacity for social-novelty recognition. This task is based on the natural inclination of rats to explore unknown social partners more than familiar ones. DAT-KO rats were used exclusively as socially atypical partners in this recognition test. Their abnormal behavioral profiles—marked by reduced affiliative engagement and compulsive traits—served as a mean to evaluate how focal rats (SX and SIKK) detected and responded to social deviance. Importantly, this design also allowed us to explore whether SIKK rats might show increased attraction towards DAT-KO individuals, possibly due to a perceived similarity or shared behavioral features.

The very first test, at younger age, was the SLAP task [22], namely a passive avoidance learning task, where water-deprived rats learn to drink only during “safe” phases (light and sound off), to avoid electric shocks associated with drinking during “unsafe” phases (light and sound on). This task allowed the measurement of the ability to form a Pavlovian link for environmental cues, predictive of safety and punishment, and to inhibit the drive to drink accordingly.

Based on previous literature and our preliminary findings, we expected that the control SX rats would perform well in all tasks, showing rapid learning in the SLAP paradigm, consistent social preferences, and a robust novelty recognition response. In contrast, SIKK rats, due to their potentially altered epigenetic background and hypothesized prefrontal dysfunction, were predicted to show a series of symptoms:

- Faster but more rigid acquisition in the SLAP task (possibly reflecting hyper-vigilance or harm avoidance or inflexible coping).
- Disorganized or absent elicitation of social preferences (directed to the SIKK social stimulus by the WT focal rat) in the elicited preference test.
- Impaired recognition of social novelty in the social recognition task.

Altogether, these paradigms allow an integrated assessment of prefrontal-dependent functions, like top-down inhibitory control and fine-grain sociability. The data allowed us to ascertain whether SIKK rats were characterized by trans-generationally inherited traits or not. The results allowed us to understand how stress during infancy, epigenetically transmitted, affects behavioral trajectories even in the great-grandsons of the stress perpetrator. Overall, our approach provides a valuable model for pathogenic pathways towards psychiatric vulnerability, even in the absence of genetic mutations.

## 2. Methods

### 2.1. Experimental Subjects

The colony of DAT-KO rats (obtained by silencing the SLC6A3 gene, which encodes the dopamine transporter) was created by inserting a stop codon that produces a truncated protein [23] at the Italian Institute of Technology (IIT, Genoa, Italy). It was maintained using a classic breeding model (heterozygote × heterozygote) for over ten generations. Some progenitors were then sent to the Istituto Superiore di Sanità (ISS, Rome, Italy), where male DAT-KO rats were crossed with Wistar-Han WT females (Charles River, Calco, Italy), producing a zero-generation (G0) of new heterozygous progenitors, referred to as MAT-HET rats. From this point onward, in subsequent generations, the breeding model used was DAT-KO male × either WT or DAT-HET female [12,24].

Offspring of DAT-KO males × DAT-HET females were termed MIX-HET [25]. A previous experiment [14] generated a peculiar K-MAT rat, by fostering MAT pups (from their natural dams, WT females) to DAT-KO females (who gave birth themselves within the previous 24 h). Interestingly, an obsessive kind of maltreatment was carried out by DAT-KO adoptive females towards K-MAT male and female F1 pups (G2). The MIK offspring at F2 (G3) were obtained by breeding K-MAT females × DAT-KO males, plus the MIX offspring came from MAT females × DAT-KO males (as the control) [14].

As a last step, at F3 (G4), SX rats are the offspring of MIX fathers (from whom they inherit the DAT trunk-mutated allele) × WT mothers [12], while SIKK rats are the progeny of MIK fathers, who are genotypically identical to MIX rats but were developing as eggs in the uterus of the K-MAT females, at time of their infancy trauma (note: the X has been replaced by K to denote the possible consequences of maltreatment), × WT mothers. 

The mothers of both SX and SIKK rats are all WT females; the MIK fathers, unlike the MIX controls, have a history of maltreatment, operated by the DAT-KO great-grandmother over the K-MAT grandmother when she was an infant, with the egg (later becoming a MIK father-to-be) still developing within her uterus. Related epigenetic markers, by consequence, could transmit these sequelae to the SIKK offspring. The comparison between SX and SIKK allows us to evaluate the inheritable effects of altered maternal care, as possibly expressed in the fourth generation within a heterozygous DAT-genotype offspring (see Figure 1). 

The animals were housed in Makrolon^®^ III cages, two per cage, matched by epigenotype, with food and water available ad libitum. The experimental room was climate-controlled (temperature: 21 ± 1 °C, relative humidity: 60 ± 10%) and maintained on a reversed light/dark cycle, with lights off at 9 a.m.

### 2.2. Setup for SLAP Experiment

The first phase of the experimental project took place at ISS, where 14 male rats (7 SX and 7 SIKK), in adolescence (21 days old at the start of the procedure and 35 days old at the end), were tested using our recently developed task [22]. 

The apparatus consisted of two Skinner box chambers (Coulbourn Instruments, Allentown, PA, USA), where individual rats were free to move, with locomotor activity (i.e., midline crossing, away from the operant side wall) measured by interruption of infrared beams. This chamber has an electrified grid floor, which can release electric foot shocks (1.5 mA; 2 s) to the animal. On the side wall, there are two nose poking devices, with a green light as a nose poke cue; the wall provides different contextual stimuli such as light (one white house lamp, located centrally on the wall) and sound (3000 Hz; 75 dB). The water dispenser is connected to a lickometer (PRS Italia, Rome, Italy).

#### 2.2.1. Preparation for SLAP Task

Three days before testing started, individual animals were put in the Skinner box chamber for habituation, with all devices off; they were left free to explore the environment and to drink ad libitum for half an hour. After this habituation day, in order to increase their motivation to drink, animals were water deprived in the home cage on the day before the test started, and the main source of water was through the Skinner box on test days. After returning to the home cages, water bottles were provided for 1 h and fluid consumption was measured. Food was always present in the home cage. Test and non-test days alternated following the schedule described below. Animals were housed in pairs within each home cage, and were tested at the same time in either of the two available Skinner boxes; they were put daily in the upper- or lower-level Skinner box (positioned on shelves of a rack), alternatively. 

#### 2.2.2. Procedure for SLAP Task

This operant SLAP task could occur with two different protocols, of which we used the simplest, called “Flash” [22]. It is based on the hypothesis that rats could be trained to drink only under a specific contingency. Specifically, rats are free to drink when light and sound are turned off. Animals should refrain from drinking under the opposite setting, when light and sound are on. In this protocol, therefore, an “unsafe” phase is characterized by the house light and sound both on, while a “safe” phase is signaled by dark and silence, plus a green nose poke cue (in one hole) turned on. One minute of unsafe phase is followed by 5 min of the safe phase and so on, alternatively, during a whole session of 30 min. If animals drink during the unsafe phase, they receive an electric shock via the electrified floor, and the 1 min of unsafe phase starts again. To get to the next safe phase, animals shall simply refrain from drinking. In this protocol, animals cannot stop the unsafe phase but (if they drink) can extend its duration (a form of punishment). Therefore, as in the “passive avoidance” schedule, animals shall simply learn not to drink when the sound and house light are on.

In such a protocol, the total duration of the safe and unsafe phases strictly depended on the rats’ behavior. The entire SLAP protocol was run for a total of two weeks, intended to train rats to drink only with the dark-and-silence predictive contingency: an automatic switch occurred between the unsafe and safe phase, unless they drank beforehand. Thus, in summary, the schedule was run for two weeks, comprising six nonconsecutive daily sessions in total (three sessions in the first and three sessions in the second week), i.e., on Monday–Wednesday–Friday.

#### 2.2.3. Water Restriction Regime

Subjects were tested from 10 a.m. to 4 p.m., for 30 min per session: within these thirty minutes, the safe and unsafe phases alternated, automatically (see above). During the weekend between Friday evening and Monday morning, when no tests were performed, the subjects were provided with 36 h of water and food ad libitum in the home cage (10 a.m. on Saturday until 10 p.m. on Sunday), to get them ready for the following Monday. Water deprivation (48 h in-between sessions) occurred for inducing a high motivation towards drinking at the resumption of test.

### 2.3. Further Experimental Subjects

The second phase of the experimental project took place at the Institute for Advanced Biomedical Technologies (ITAB), University “Gabriele D’Annunzio”, Chieti. Rats were transferred from ISS to ITAB under veterinary authorization and surveillance.

A total of 16 male rats (8 SX and 8 SIKK) were tested. The SX and SIKK rats represent a fourth-generation heterozygous rat model for the epigenetic sequelae, potentially inscribed onto the gene encoding the DAT transporter. To realize social encounters, 4 WT rats, and 7 DAT-KO rats (also coming from ISS) were used.

#### 2.3.1. Setup in Common for Both Social Experiments

The experimental encounters were carried out using a setup consisting of a plexiglass box with two connected rooms. Both rooms (30 cm × 35 cm × 40 cm) had grey walls and floors, the back wall of one chamber was white, while that of the other chamber was black [26]. Each room was equipped, in one corner, with a small metal cage with vertical grey bars (20 cm × 20 cm × 50 cm), which could either be empty or contain a confined stimulus rat. Meanwhile, an explorer “focal” rat was free to move between the two rooms, through a central opening. DAT-KO rats were not included as experimental subjects but were used as socially deviant stimuli, based on their well-documented alterations in social behavior and reduced affiliative interactions. This setup allowed us to assess the ability of focal rats (SX and SIKK) to discriminate between (and be discriminated by) typical (WT) and atypical (DAT-KO) social partners, thereby probing their social sensitivity and affiliative preferences (both expressed and elicited). 

The focal rat’s exploratory behaviors were monitored using a video camera mounted on a tripod, positioned above the apparatus to obtain a top-down view. The observation sessions were subsequently analyzed to quantify the time spent in each room, the number of entries, the escape attempts, and the interactions of the focal rat (free to move within the whole environment) with either environmental items or the stimulus rats (in order to evaluate preferences and social recognition abilities).

Two different social tests were performed. The first test examined the preference for social interaction of a wild-type (WT) focal rat, aiming to evaluate its preference for interacting with a DAT heterozygous stimulus rat versus spending time in an empty room (devoid of social stimuli). Of course, the social preferences and exploratory behavior of the WT rat could vary in the presence of a social stimulus (either SIKK or SX epigenotype) compared to a neutral environment.

The second test explored the social recognition ability of the SIKK vs. SX focal epigenotypes. We tested the decision-making process behind the choice between two stimulus rats (one WT and one KO) as well as the recognition of a social change (following the habituation of heterozygous focal rats to the continuous presence of the same DAT-KO stimulus rat). During the first three days, SIKK and SX rats were exposed to the same KO stimulus rat while, on the fourth day, the KO rat was replaced with another, previously unknown, KO rat. This experiment aimed to determine whether SIKK and\or SX rats could effectively detect the social change, based on their reactions when faced with a new individual. 

#### 2.3.2. Elicited Preference Experiment

The cohort for the first test consisted of 20 rats (4 WT rats, 8 SX rats, and 8 SIKK rats).

The day before the start of the protocol, all rats underwent a “habituation” period, during which each focal animal was individually placed inside the apparatus for 15 min, with the opportunity to explore the whole environment and the empty cages. Similarly, each stimulus rat was placed individually within the metal cage for 15 min, to get used to be confined within the little space available. The rats’ behavior was observed from Monday to Thursday for two weeks, with observations divided into two daily time-windows (10:00–12:15 and 13:30–16:30).

Each morning, the whole cohort of rats was transported from the facility to the experimental room, where behavioral observations were conducted, and left undisturbed for a 30-min period before proceeding with the actual test.

The experiment involved a social encounter between a SIKK/SX rat, positioned as a stimulus inside the cage in one of the two rooms of the apparatus, alternately, and a WT focal rat, which was placed in the opposite room as a starting point and allowed to move freely throughout the environment. All focal rats encountered all possible stimulus rats. The total duration of each social encounter was twenty-five minutes. The focal rats’ behavior was recorded using a video camera that operated for the entire daily duration of the experiment.

Following each social encounter, the rats were returned to their home cages (with water and food provided ad libitum), and the walls and floor of the apparatus were cleaned with an alcoholic solution (50% *v*/*v*) to prepare for the next session. The behaviors analyzed included exploratory activity, cage sniffing, cage rearing, wall rearing, self-grooming, allogrooming, and inactivity with positioning either near or far from the metal cages.

The researchers remained in the room, minimizing any interference with the procedure as much as possible. The video recordings were saved onto a micro-SD card and later transferred to a designated cloud storage platform, where they were processed using video analysis software (The Observer XT 10; Noldus, Wageningen, The Netherlands) to extract the relevant data.

#### 2.3.3. Social Recognition Experiment

For the social recognition test, the cohort of subjects included 25 rats (4 WT rats, 7 KO rats, 7 SX rats, and 7 SIKK rats). The SX and SIKK rats were used as focal animals, while the WT and DAT-KO rats served as stimulus animals. The behavior of the focal rats was assessed in relation to the genotype of the stimulus rats.

The observations were conducted over two weeks, from Monday to Thursday, during two daily time-windows (10:00–12:00 and 13:00–15:30). On each day, all cages containing the designated rats were transported from the animal facility to the experimental room, where the testing apparatus was located. The apparatus used was the same as in the previous test.

After an initial thirty-minute rest period in their home cages, the rats were placed in the apparatus according to a pre-established schedule of social encounters. In the room with the black wall, the KO stimulus rat was placed inside its metal cage, while the WT stimulus rat was placed in its metal cage in the room with the white wall. Finally, the focal rat of either SX or SIKK epigenotype, in a counter-balanced order, was introduced near the connecting door. Focal subjects were free to explore both rooms and interact with the metal cages containing the stimulus rats, during a thirty-minute social encounter. 

**Figure 2:**
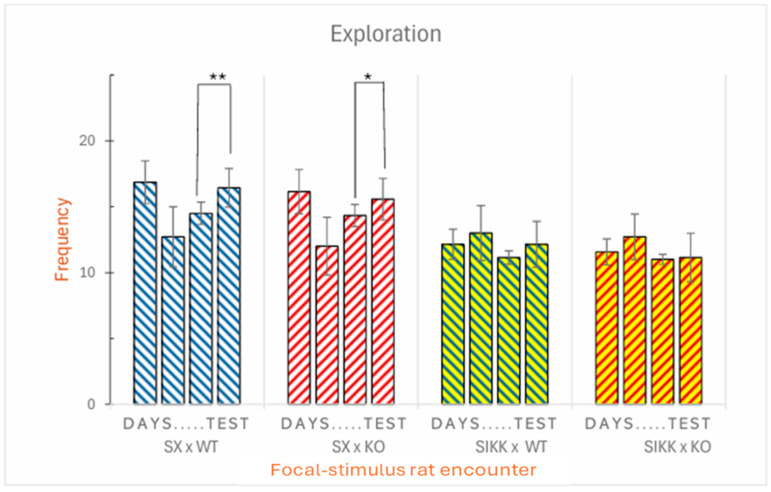
Social recognition: **frequency** of exploration focal SX and SIKK rats towards the room with WT or KO stimulus rats: mean frequency ± SEM for transitions from any other behaviors to the exploratory one shown by the focal SX and SIKK rats in the presence of WT (blue bars) or KO (red bars) stimulus rats. The bars are divided into blocks representing the days of social encounters: for three consecutive days, focal rats encounter the same stimuli, while on the fourth day (test) the KO stimulus is replaced by another KO stimulus. Between the third and fourth days, an increase in transitions from other behaviors to exploration is found in the SX focal rats towards the WT and KO stimulus rats (** *p* < 0.01 and * *p* < 0.05).

The four different WT rats were combined in a counter-balanced way with four distinct KO rats and these stimulus pairs interacted with alternating epigenotypes, i.e., with the SX and the SIKK focal rats. For three consecutive days, the encounter combinations remained identical. On the fourth day, however, the “familiar” KO stimulus rat in each interaction pair was replaced with a novel and yet unknown KO rat. The aim was to detect any behavioral modifications in the focal SIKK or SX rats, in response to recognizing this social change (see fourth vs. third day in Figure 1 and Figure 2).

As in the previous test, behavioral observations were recorded using a video camera positioned above the apparatus. The experimenters remained in the room, minimizing any interference with the ongoing procedure. The video recordings were later analyzed as described before. The behavioral items scored were the same as those of the previous test.

### 2.4. Data Analysis

Behavioral data were analyzed by repeated measures ANOVA (RM-ANOVA), using StatView II software (Abacus Concepts, Berkley, CA, USA). The significance level was set at *p* < 0.05 and post hoc analysis was performed using Tukey’s HSD test. 

The non-significant tendencies were also considered at 0.05 ≤ *p* ≤ 0.10 and confirmed by multiple post hoc comparisons carried out through the Tukey HSD test. The latter is a stand-alone test, which can be run even in the absence of significance in the main ANOVA. None of the experimental subjects had to be excluded because of outlying data. All results are expressed as mean +/− SEM.

## 3. Results

### 3.1. SLAP Results

*Licking rate per session*: Our observations showed that all the animals in both groups (SX and SIKK) exhibited a significantly higher licking rate during the safe phase (see inefficient licks), while largely avoiding licking during the unsafe phase (see efficient licks). This suggests that all the rats were able to recognize the predictive value of environmental contingencies.

Notably, SIKK rats made significantly larger erroneous licks during the unsafe phase (see efficient licks), indicating a worst understanding of the task, compared to SX rats (F_1,12_ = 5.71; *p* < 0.05). However, the amount of free drinking during the safe phase (water consumed during session, see inefficient licks) did not differ significantly between the two groups, suggesting that both were equally efficient in adapting fluid intake to the task. Note that six hundreds drops each of 20 microliters imply 12 mL of water intake, approximately.

*Locomotor escape-like activity*: SIKK rats displayed a significant tendency towards lower locomotor activity (see midline crossing towards the inactive side of the Skinnerbox) than SX rats (F_1,12_ = 3.58; *p* = 0.08): SIKK rats appeared to exhibit a slight inhibition, possibly due to the increased number of punishments received.

*Duration of the unsafe phase and number of foot shocks*: The unsafe phase tended to be shorter in SX than SIKK rats (F_1,12_ = 3.72; *p* = 0.07), suggesting a possible improvement in task learning for controls rather than SIKK subjects (Mean collective unsafe phases lasted 5′54″ for SX controls and 6′42″ for SIKK rats). In fact, the unsafe phase was extended whenever licks resulted in a punishment.

This trend was further supported by the significantly higher number of foot shocks received by SIKK rats compared to SX rats (F_1,12_ = 4.81; *p* < 0.05). SIKK rats received at least one more foot shock than controls, implying that unsafe phases lasted at least one minute more for them. 

*Water consumption*: When analyzing water intake within 30 min after returning to the home cage, we found that SIKK rats drank more than SX rats. This suggests that worst task performance was associated with an enhanced need to drink after completing the test (F_1,12_ = 6.30; *p* < 0.05) (see Table 1). Note that water drunk from bottles within 1 h after returning to the home cage was approximately half the volume already drunk during the operant session. 

### 3.2. Elicited Preference Task

To evaluate social attractive behavior in SIKK vs. SX rats, we conducted a social preference test on the focal WT subjects. The aim was to observe whether a wild-type (WT) focal rat tended to spend more or less time near or interacting with a SIKK stimulus rat, compared to the SX controls. The results obtained for the different behaviors aligned with our expectations.

Regarding exploratory behavior, we observed a significant effect both in terms of frequency and duration (F_1,48_ = 16.92, *p* < 0.001; F_1,48_ = 34.49, *p* < 0.001, respectively), as expected from previous data, reporting scarce interaction between WT and DAT-HET rats [24]. WT focal rats showed a significant preference for visiting the empty room more frequently, and for a longer duration, compared to the room containing the stimulus rat (data are consistent with those shown in [21]). Conversely, we observed that WT focal rats engaged in self-grooming more often and for longer periods than in the room with the stimulus rat (F_1,48_ = 4.80, *p* < 0.05; F_1,48_ = 11.9, *p* < 0.001, respectively).

However, the most interesting result concerns allogrooming behavior, which occurs when the focal rat grooms the stimulus rat, and the latter accepts the interaction. This behavior represents a form of mutual social interaction between the two individuals. A significant finding emerged (as an effect of the group factor: F_1,48_ = 3.78; *p* < 0.05), as the WT focal rats engaged in a greater number of allogrooming interactions with SX rats than with SIKK rats. Another similar result concerns the duration of allogrooming, for the room × group interaction (F_1,48_ = 5.38; *p* < 0.01). We observed that WT rats engaged in allogrooming for a significantly longer duration with control SX stimulus rats, while this duration was significantly reduced when interacting with the SIKK stimulus rats (data are specular to those shown in Figure 4 and Figure 5. Specifically, whereas in the EPT SIKK rats did not concede to be allogroomed by focal WT rats, in the SRT SIKK rats did not sniff the cage when a WT was inside, denoting poor solicitation towards allogrooming). As a matter of fact, SIKK rats were somewhat not attractive and possibly exhibited atypical behavior.

### 3.3. Social Recognition Task

In the course of this paradigm, the focal animal is repeatedly exposed to a stimulus animal. After several encounters, a marked decrease in the time spent by the focal animal on olfactory investigation of the stimulus animal is observed. Finally, when exposed to a new and unfamiliar stimulus animal, the focal animal’s investigation time returns to its original levels [27]. By observing the focal SX or SIKK rats freely moving inside the apparatus, we analyzed their behaviors. Observing the exploratory behavior of the focal rats, both in terms of frequency and duration, we can conclude that the social recognition test was effective and reliable.

*Exploring. Frequency:* The analysis revealed significant differences between the two groups of rats. Both groups of focal rats spent more time exploring the room containing the WT stimulus rat compared to the room with the KO stimulus rat, as indicated by the stimulus × genotype interaction (F_1,48_ = 7.35; *p* < 0.01). Additionally, SX focal rats explored more frequently than SIKK focal rats, nearly a 50% more.

However, a more in-depth analysis using Tukey’s post hoc test yielded very contrasting results. Indeed, on the test day (when the KO stimulus rat was replaced with a new, unfamiliar KO rat), SX rats tended to inspect the room containing the DAT-KO rat more frequently, as expected. Control SX rats also tended to explore the room with WT rats a bit more frequently on test day. This observation suggests that control SX rats clearly noticed the change; yet, to some extent, they also re-explored the WT stimulus which was not changed (see Figure 2, left half).

No significant difference was observed in the exploratory behavior of SIKK rats towards the KO or WT stimulus (*p* > 0.05) between the third and fourth day (see Figure 2, right half).

*Exploring. Duration:* Using repeated measures ANOVA, a significant interaction effect was found between focal genotype × stimulus epigenotype × day (F_1,48_ = 2.931; *p* < 0.05). Specifically, focal SX and SIKK rats as a whole spent more time exploring the room containing the KO stimulus rat, particularly on the test day, i.e., when comparing the third to the fourth day. Now, to further investigate potential differences between the two epigenotypes of focal rats (SX and SIKK) in their ability to recognize a change in either genotype (WT or KO) of stimulus rat, we conducted Tukey’s post hoc test. These analyses revealed some interesting findings.

**Figure 3:**
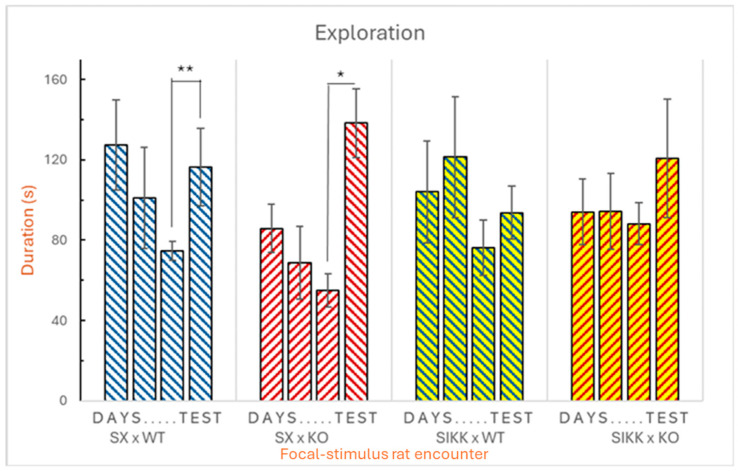
Social recognition: **duration** of exploration focal SX and SIKK rats towards the rooms with WT or KO stimulus rats: mean duration ± SEM of exploratory behavior shown by focal SX and SIKK rats in the presence of WT (blue bars) or KO (red bars) stimulus rats. A significant difference is highlighted between the third and the fourth day, in the control SX group, with an increase in the duration of exploration towards the WT stimulus rats (** *p* < 0.01) and towards the KO stimulus (* *p* < 0.05). Like already evident for frequency, no significant difference is observed in the duration of exploratory behavior shown by SIKK rats towards the KO stimulus between the third and the fourth day.

Notably, focal SX control rats were more sensitive to the change that occurred on the fourth day, showing a significant increase in the duration of exploration within the room containing the new KO stimulus rat. Additionally, a marginally significant increase for time spent in the room containing the WT stimulus rat was also observed (see Figure 3, left half). Conversely, no significant difference was found in the exploratory behavior of SIKK rats towards either the WT or the KO stimulus, between the third and fourth day (see Figure 3, right half).

Regarding allogrooming and cage sniffing, we obtained contrasting results.

**Figure 4:**
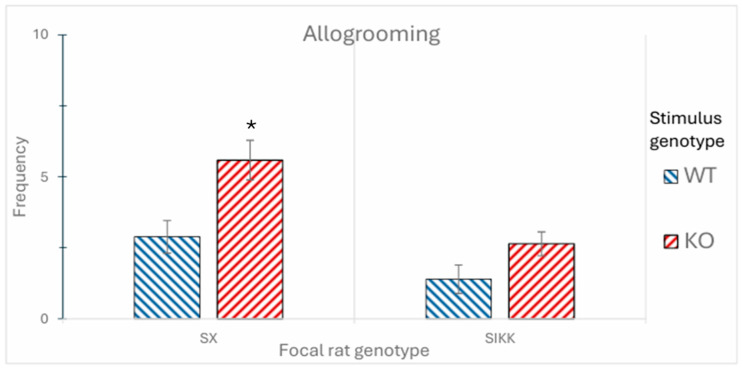
Frequency of “**allogrooming**” in interaction with different stimuli (WT or KO) shown by SX and SIKK focal rats. Data are presented as mean ± SEM number of allogrooming episodes performed by SX and SIKK rats towards WT (blue bars) or KO (red bars) stimulus rats. A significant trend is observed for control SX focal rats, which interact more frequently with KO rats (despite anomalous behavior) than with WT stimulus rats. This affiliative profile is almost absent in SIKK rats. * Indicates a significant difference between KO and WT stimulus rats, within control SX group (*p* < 0.05).

*Allogrooming. Frequency*: When examining allogrooming behavior between rats, a significant difference was observed between the two stimulus genotypes (F_1,48_ = 14.06; *p* < 0.001), which translates into a greater frequency of reciprocal interactions between SIKK and SX focal rats, taken as a whole, with KO rats compared to WT rats. Unexpectedly, KO stimuli were prone to accept allogrooming by focal rats (see Figure 4). Furthermore, in the stimulus epigenotype × focal genotype interaction, explored by Tukey’s post hoc test, a trend toward significance was observed for control SX focal rats, with the anomalous DAT-KO rats interacting more frequently (by accepting grooming) than the WT rats. This condition was not observed in SIKK rats, which displayed overall very low levels of allogrooming.

**Figure 5:**
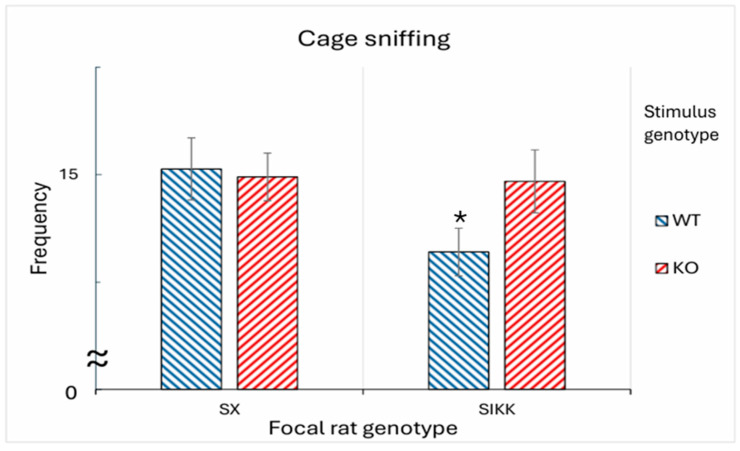
Frequency of “**cage sniffing**” exhibited by focal SX and SIKK rats directed at the metal cages containing either WT or KO stimulus rats. Data are presented as mean ± SEM number of sniffs directed at the cage housing a WT (blue bars) or KO (red bars) stimulus rat. Focal SX rats do not show a significant difference in sniffing frequency, regardless the genotype of stimulus rat (WT or KO) present in the cage. In contrast, focal SIKK rats exhibit a reduced frequency of cage sniffing when the stimulus rat is of WT genotype. * Indicates a significant difference between WT and KO stimulus rats, within SIKK group (*p* < 0.05).

*Cage-sniffing. Frequency*: Consistent with the findings on allogrooming behavior, cage sniffing yields somewhat specular results, specifically in the stimulus epigenotype × focal genotype interaction (F_1,48_ = 3.30; *p* < 0.05). The Tukey post hoc analysis suggests a significant picture: the SIKK focal rats tend to sniff the cage of the WT stimulus rat for a shorter time (see right half of Figure 5) than the one containing the KO one. These observations seem to suggest that the SIKK rats notice a genotype-related change in the behavior expressed by either stimulus, and try to solicit an interaction with the KO ones; meanwhile, the SX control rats do not show a preference between the two genotypes.

## 4. Discussion

As previously described, the colony of DAT heterozygous (HET) rats, whose behaviors we observed, originated from crosses that differ from classical HET × HET breeding: rather, we adopted a biased mating between a completely wild-type (WT) dam and a completely mutated (DAT −/−) sire (or vice versa) as progenitor lines [8,9,10,11]. These crosses produced biased heterozygous rats (called MAT) that differed from classical HET offspring [24,28]. When bred with KO rats, MAT females gave rise to a second-generation offspring, identified as MIX [12]. Interestingly, a parallel line in our colony was made of MAT pups that were fostered, at birth, to KO females [14]. The obsessive care of the latter dams generated an infantile trauma in the MAT females; hence they were named k-MAT. When these females gave birth, in turn, their offspring (termed MIK instead of MIX) were hyperactive and completely inattentive [14]. We here analyzed the behaviors of the fourth-generation offspring: we hypothesized that their anomalous phenotype might stem from two possible sources. The first and more obvious is the direct impact of maternal behavior, displayed by k-MAT dams. A second more subtle possibility is that the maltreatment, suffered by their k-MAT parent when infant, and due to the anomalous maternal care provided by the DAT-KO grand-dam, could have somewhat an epigenetic impact on the developing gonads. Namely, the uterus and eggs of k-MAT developing subjects, which later in life would host the fetus of MIK males, could possibly receive an early epigenetic imprint.

In the present research project, we studied one further generation of heterozygous rats that would inherit such a hypothesized epigenetic marker, possibly transmitted through their father’s sperm. In the case of SIKK rats, these putative epigenetic effects derive from two sources: the history of maltreatment (operated by the DAT-KO great-grandmother over the infant, lactating grandmother k-MAT) was experienced by the SIKK-offspring father, i.e., the MIK, not only (1) under the form of altered maternal care (displayed by k-MAT females once they become dams), but also (2) well before, at the time when the MIK was just a developing gamete (i.e., an egg, inside the developing uterus of k-MAT pups). 

Being heterozygous for DAT gene, the SIKK rats and their SX controls carry a wild-type allele and a mutated one (which has been artificially truncated by inserting a stop codon at the beginning of the sequence) [23]. As a result, several behavioral anomalies have already been observed in DAT-HET rats, depending on the lineage of truncated DAT protein mutation [12,24,28]. Yet, additionally, SIKK rats may inherit (via the sperm of their sire) a DAT mutated allele which also has putative epigenetic marks of the aforementioned transmission of trauma. In this context, wild-type (WT) rats represent the healthy baseline, while DAT-KO rats show pathological extremes, such as hyperactivity, disorganized and stereotyped behavior, and reduced affiliative tendencies. Our DAT heterozygous lines (SX and SIKK) fall genetically in-between these two extremes. 

Interestingly, behavioral variability within the DAT-HET population is frequently observed: some individuals resemble the WT phenotype, while others exhibit patterns closer to those observed in DAT-KO rats. This distribution suggests that non-genetic, possibly epigenetic, factors—especially those linked to early trauma in the lineage—may modulate the phenotype of these heterozygous individuals, pushing them toward either a resilient or vulnerable behavioral profile. The aim of the current study was to determine the epigenetic differences transmitted across three generations, by comparison between SX and SIKK rats. These differences would allow us to evaluate only the transgenerational impact of trauma, devoid of maternal influences. since both groups of rats have been raised under normal maternal conditions by WT dams.

### 4.1. SLAP Task: Predictive Cues and Avoidance

Learning to avoid aversive outcomes is an adaptive strategy to limit one’s future exposure to stressful events. In the “Flash” SLAP protocol, all rats demonstrated the ability to differentiate the safe from the unsafe phase, progressively reducing licking during the latter. However, the SIKKs showed significantly slower learning, eventually making a greater extent of errors, than the SX controls. This hyposensitivity to negative stimuli may result from an altered regulation of the dopamine system, leading to decreased reactivity to threat. Therefore, while SX controls quickly displayed a passive avoidance and got significantly reduced foot shocks in the unsafe phase, the SIKK rats did not. This evidence can be interpreted so that the SIKKs perceive the punitive stimulus as less aversive than SX controls do. In other words, they could be less sensitive to negative reinforcement, or less flexible to adapt, hence developing a smaller reactivity to aversive stimuli.

Exposure to aversive stimuli can elicit a range of responses across a population, with some individuals that readily learn to avoid the negative outcome whereas others do not. Behavioral differences could arise from intrinsic differences in the neural response, during aversive events, in circuits mediating reinforcement and motivated behavior. The neurotransmitter dopamine is thought to regulate behavioral responses to aversive stimuli, through the mesolimbic dopamine system, in reward-based tasks. In support, stimulating dopamine neuronal activity improves the active avoidance performance [29].

Initially, dopamine responds directly to reward but, as learning progresses, dopamine release shifts to the predictive cues of the expected outcome. Dopamine in fact conveys a prediction error signal, representing the difference between the expected and actual reward. This mechanism functions similarly in positive (rewarding) and negative (aversive) tasks: dopamine response should shift from the aversive stimulus itself to its predictor, and encode a safety prediction error. However, the exact dynamics of dopamine signaling in avoidance learning remain unclear [29]. The present study used the SLAP paradigm (based on a passive avoidance task) to investigate behavioral differences between SX and SIKK rats. Our particular attention was devoted to the effects, in DAT-HET rats, of a possible transgenerational transmission of trauma.

Dopaminergic activation within the ventral tegmental area (VTA) and nucleus accumbens (NAcc) facilitates the association between a neutral stimulus and an aversive event, reinforcing fear memory. D1 and D2 receptors in the amygdala are essential for modulating the fear response: blocking these receptors reduces the acquisition of fear conditioning [30]. Studies on animal models have shown that the administration of D2 antagonists in the basolateral amygdala reduces the freezing response in rats [31]. Hence, D2 receptors are crucial for the expression of conditioned fear. Inhibition of dopaminergic transmission in the mesolimbic pathway can reduce the potentiation of the fear response. Studies indicate that cortico-mesolimbic dopamine neurons fire in response to conditioned stimuli, and transmit signals to the prefrontal cortex, amygdala, and ventral striatum.

In particular, reduced self-control activity in the prefrontal cortex is associated with greater rigidity and impaired adaptation to fear generalization. Epigenetic silencing of DAT, with heightened dopaminergic tone and impaired phasic release, may explain why SIKK rats showed a less intense adaptive response to aversive stimuli. Interestingly, SIKK rats descend from a line where both the grand-dam and the sire were subjected to early-life maltreatment and gamete-level stress, respectively. These alterations were transmitted to SIKK offspring via the sperm of the MIK sire and contributed to impaired sensitivity to aversive stimuli. Such studies suggest that transgenerational alterations in dopamine function influence individual response to threatening stimuli.

### 4.2. EPT, Conducted with WT Focal Rats

In the EPT experiment, we aimed to analyze the behavior of WT rats toward SX or SIKK rats, placed as stimuli within the metal cages. We sought to investigate differences in the social behavior of wild-type, normotype rats towards control SX rats compared to potentially anomalous SIKK rats. The goal was to determine the reaction elicited by SIKK rats; namely we observed whether WT rats were attracted to them or, conversely, exhibited any avoiding of such anomalous social stimuli. Indeed, epigenetic inheritance of trauma could alter the social signals expressed by SIKK rats, whose social skills have likely been shaped inter- and trans-generationally [32].

Our analysis shows that WT focal rats tend to spend significantly more time in the empty room (without stimuli) and less time in the room containing the SX/SIKK stimulus. This is in line with our previous results about the poor interaction of WT with DAT-HET rats [21,33,34]. However, at the same time, they tend to sniff the cage containing the stimulus more frequently than the empty one, suggesting residual curiosity. WT rats seem to have a greater preference for interacting with the SX control rat and appear less interested in interacting with the SIKK anomalous rats. Despite being DAT heterozygous (like the SX rats) and having received normal maternal care (since both groups were raised by WT mothers), SIKK rats clearly exhibit some social signals that WT focal rats perceive as abnormal. This suggests that transgenerational inheritance has occurred. The elicited preference test allowed us to assess that epigenetic trauma has been transmitted to the fourth generation. 

We propose that epigenetic alteration has modified the behavioral, social, or olfactory signals expressed by SIKK rats, making them perceivable as “anomalous” or “different” (at least compared to standard DAT-HETs, like SX rats). Since WT rats are genetically neutral and behaviorally normal, their preferences (i.e., reactions like approaching or staying away) do reflect a spontaneous response to the presented social stimuli: SIKK rats turned out to be affected by sociability biases, related to previous pedigree trauma. These findings are consistent with a very recent study that highlighted the influence of transgenerational inheritance: the offspring of DAT-HET rats, that had inherited epigenetic modifications from a QULL father or a QULL maternal grandfather, showed lower sociability than other groups [13]. This suggests that the inheritance of epigenetic traits from grandparents can negatively influence social behavior, leading to behavioral alterations compared to controls [11,12,13].

### 4.3. SRT, Conducted with SIKK or SX Focal Rats

Social recognition tests are used to examine learning and memory behaviors, originally developed as a non-aversive alternative to traditional avoidance paradigms [35]. These tests rely on the natural tendency of animals to investigate the presence of new conspecifics through smell. Genital and head sniffing, as well as following closely, are considered investigative behaviors.

In the social recognition test, we used SX and SIKK rats as focal rats, and WT vs. KO rats as stimulus rats. The aim was to detect differences in the interest of focal rats toward WT stimulus rats versus clearly anomalous DAT-KO rats. Additionally, we sought to identify cognitive differences between focal rats in their ability to recognize that a change occurred in the KO stimulus rat on the fourth experimental day.

It clearly emerges that the exploratory behavior of the focal rats shows a preference for the WT rat, both in frequency and duration. However, on the last schedule day, when the KO stimulus rat was replaced with another, yet unknown one, SX focal rats recognized the change and visited the KO stimulus more frequently, whereas SIKK rats did not seem to notice the change. Specifically, it appears that SX and SIKK rats show dramatic differences in social recognition ability: the SIKK rats appear devoid of a social recognition capability, probably related to their epigenetic history and inherited transgenerational influences.

The prenatal phase is essential for epigenetic changes. Recent studies have proposed that when a “virgin” allele (i.e., from a pure WT lineage) first encounters a DAT null (KO) allele upon formation of the zygote, an intra-allelic epigenetic process may occur in MAT heterozygous zygotes. As such, the wild allele is somewhat “marked”, and sequelae of this become apparent in the following generations [10,11,12,24]. However, data suggests that such an alteration only affects the first generation i.e., the MAT rat and (in the opposite way) the second generation i.e., the MIX rat. Afterwards, in the subsequent generations, the putative epigenetic modification no longer has observable effects on the phenotype [12,14]. The results of our current study indicate that SX epigenotype rats show behavior similar to that of WT rats, meaning that they display correct recognition of the DAT-KO stimulus change. This suggests a return to behavioral normality.

As described in Section 4.2, allogrooming and cage sniffing behaviors show apparently contrasting patterns. SX focal rats appear to engage in allogrooming more frequently with the KO than with the WT stimulus rat, while SIKK focal rats tend to sniff KO rats more than WT ones. These observations align with what previously found [33], with DAT-KO stimulus rats reported to be more attractive than other stimuli, perhaps because they move continuously and compulsively (despite being inside a small cage). While cage sniffing consists of poking the nose between metal grids of the cage, to reach the stimulus rat’s body, the stimulus rat could well remain apparently uninterested in that. Conversely, allogrooming involves the focal rat actively interacting with the stimulus rat in a way that implies a permissive (and hence reciprocal) response from it.

Thus, it seems that SIKK and SX focal rats are both interested in the stimulus rats, but they can only engage in allogrooming with those that reciprocate their interest. The fact that control SX rats engage in allogrooming with KO rats suggests that KO rats actively accept interaction with SX rats. In contrast, SIKK rats show little allogrooming, overall. This can be due (1) to their own poor inclination to provide care to WT or KO rats, and/or (2) to WT or KO rats being not, in turn, inclined to receive care from SIKK rats. As far as WT stimuli are concerned, it appears that the SIKK rats do not have a prosocial inclination of their own. Looking at the cage sniffing behavior, SIKK rats show a heightened interest in KO rats, suggesting their potential propensity or desire to interact with them; possibly, SIKK rats feel somewhat similar to KO rats. However, KO rats do not seem to accept such solicitation, showing little interest in receiving an allogrooming by SIKK rats. This picture could again be explained by a perception of anomalies, with DAT-KO rats still capable of recognizing some atypical social characteristics in SIKK rats. 

### 4.4. Comparison with the State of the Art

The role of epigenetics is crucial [36,37] in transgenerational inheritance. Such a notion must be subject to two conditions: (A) the presence of an epimutation in the great-grandparents is associated with the specific disorder in the grandparents and then in the subsequent offsprings; (B) the inherited epimutation in the offspring is associated with the risk of phenotypic pathology [38]. In other words, there must be at least three-level traumatic event (genetic vulnerability + environmental adversity + epigenetic imprint) in the F0 generation, and the sequelae of the event must be observable in at least three subsequent generations [39,40]. It follows how, in epigenetic transgenerational inheritance, epimutation of grandparents can develop along the germ line, beside somatic cells, producing phenotypic changes [41,42] and leading to the consolidation of phenotypic changes via the epigenetic marker’s inheritance [43].

Maternal behavior permanently alters HPA responses through classical effects on gene expression. Experience of stress during early childhood alters social and emotional traits, learning and memory formation in rodents [44,45,46,47,48,49]. The excess or the absence of maternal care during early childhood alters the hypothalamic, serotonergic, and dopaminergic systems [50,51]. During chronic and acute stress, the release of corticotropin (CRH) stimulates the secretion of adrenocorticotropic hormone (ACTH), which causes the release of cortisol from the adrenal glands. The latter binds to the glucorticoid receptor (GR) and activates the NR3C1 gene [52]. Accordingly, maternal-behavior variations exert a crucial role in non-genomic transmission mechanism, promoting individual differences in stress reactivity between generations [53]. Variation in maternal care alters the methylation status of the exon 17 promoter of the GR gene; the maternal effect on F1 is also mediated by alterations in chromatin structure [53], hence leading to stable alterations in phenotype. It is tempting to speculate that such molecular changes occurred in our rats. 

The current grand-offspring (i.e., F2) gets inheritance of traits depending on non-genetic modifications in germ cells occurring within F1 in infancy. Functional consequences develop in F2 grand-offspring both during neuro-development and in adulthood [14]. Current data confirm that such behavioral changes may be further inherited by subsequent generations [3,19]. Of note, epigenetic markers in germ cells are influenced by environmental factors. Male gametes are most vulnerable in the early pre- and post-natal stages, due to the expression dynamics of epigenetic markers [3,19].

### 4.5. Limitations and Key Summary

A key limitation of our study is the absence of direct molecular evidence for the proposed epigenetic mechanism. While our findings are consistent with a biological transmission of early-life trauma, we cannot yet confirm whether this occurs via epigenetic modifications such as DNA methylation or non-coding RNAs. Further molecular analyses will be essential to validate this hypothesis. Importantly, we intentionally used only male subjects—both as offspring and as transmitting parents—to exclude any contribution of maternal behavior or social learning. 

Unlike females, male rats do not raise or interact with their offspring, meaning that SIKK phenotype cannot result from behavioral imitation or trauma repetition. Therefore, what is transmitted must occur biologically, not through environmental or relational factors. Sires did not act behaviorally nor relationally onto their offspring: it is unlikely that the anomalous MIK phenotype, compared to MIX controls [14], affected the future maternal behavior of WT dams after just a couple of weeks of mating interaction. While it is possible that MIK sires developed certain traits behaviorally, due to their own upbringing [14], the fact that SIKK rats were raised exclusively by wild-type dams eliminates direct social and/or environmental transmission. Thus, our data strongly suggest a germline-based mechanism, though its epigenetic nature remains to be formally demonstrated.

In summary:

- SX rats represent evidence that anomalies displayed in the first (F1: MAT) and second (F2: MIX) generations are over: they seek stable and predictable relationships, approaching WT rats as they are behaviorally normal, but still maintain a genuine curiosity towards DAT-KO rats, due to an instinctive exploratory drive towards novel or unusual stimuli.
- SIKK rats, on the other hand, show a subtle predisposition towards dysfunctional or irregular social patterns, with poor social recognition; they are somewhat avoided by WT (simple preference) and by KO (refusal to be allogroomed) rats. This is potentially linked to influence of unresolved ancestral traumatic experiences. This piece of data clearly reflects the inheritance of epigenetic signals deriving from grandmothers (who lived their infancy in hyper-stimulating and stressful contexts, deriving from obsessive care received by DAT-KO great-grandmothers).

## 5. Conclusions

Despite SIKK rats never having directly experienced any maltreatment or stressful conditions, the negative inclination of the other genotypes against them could be linked to an antisocial display, possibly with somewhat obsessive–compulsive traits, transmitted epigenetically from their ancestors. In particular, sequelae propagated via the gametes of their MIK sires, which did develop as eggs in the uterus of the maltreated k-MAT grand-dam. KO rats, with their extreme and compulsive hyperactivity, represent a “familiar” behavioral model that SIKK rats seem to solicit socially; yet, KO rats do not find it comfortable to be allogroomed by them. 

This suggests that epigenetic transmission influences not only major behavioral parameters but also social preferences and social relationship patterns, and that its effects extend well beyond the first generation. In fact, as discussed above, for an epigenetic mechanism to be considered a plausible cause of a pathological phenotype, epigenetic changes and the resulting phenotype must persist into the fourth generation and beyond [39,40]. This criterion is based on the fact that a female’s exposure to gestational stress directly affects not only the developing embryo, but also its own developing uterus (or testes) and germline. As a result, phenotypic alterations observed in the first (F1) and second (F2) generations may stem from a direct exposure to the environmental stressful factors, rather than from true germline transmission. In the subsequent F3 generation, we are certain that nothing was there at the time of the trauma. The MIK sire was there as an egg within k-MAT infant females, but paternal gametes, i.e., MIK sperm later originating zygotes for SIKK pups, did not yet exist at the time of the original trauma.

These findings can be interpreted in light of the role of transgenerational epigenetic modifications in shaping self-control and social behavior, as well as social empathy [54]. Epigenetic memory, inherited through mechanisms such as modifications in DNA methylation or non-coding RNAs, may preserve traces of ancestral experiences of stress and abuse. This could influence the social and relational choices of subsequent generations, pushing them towards behaviors and interactions that reflect ancestral social dynamics, having occurred between the great-grand-dam and the grand-dam, as presently demonstrated. 

## Figures and Tables

**Table 1:** Behavioral and water-consumption data of rats with different epigenotypes (SX vs. SIKK) during the SLAP protocol.

Variables	Experimental Groups		
	SX		SIKK
Efficient licks during unsafe phases	2.46 ± 0.32	*	3.82 ± 0.47
Inefficient licks during safe phases	23.05 ± 3.41		26.36 ± 3.02
Inactive behavior (midline crossing)	88.89 ± 9.01	*	70.07 ± 4.23
Cue-2 nosepoking responses	7.80 ± 0.24		8.46 ± 0.24
Shocker	1.86 ± 0.29	*	3.21 ± 0.55
Amount of water consumed	6.98 ± 0.20	*	8.70 ± 0.65
Amount of food consumed	2.68 ± 0.16		2.50 ± 0.05

The table reports mean ± SEM for each variable: efficient (to trigger a foot shock, i.e., during unsafe phases) and inefficient (to trigger a foot shock, i.e., during safe phases) licking responses (25 drops per lick), inactive-side behavior (frequency of midline crossing, towards the inactive side of the Skinnerbox), Cue-2 (nose poking during unsafe phases) responses, shocker (number of foot shocks received), and total water (mL) and food (g) consumption, after returning to the home cage (within 1 h). SIKK rats showed higher punished errors via efficient licking (i.e., during the unsafe phases), greater water intake, and increased foot shock received, suggesting a divergent behavioral profile compared to SX controls. * Indicates a significant difference between SX and SIKK group (*p* < 0.05).

## Data Availability

Data are stored on a PC in the office of the corresponding author and can be shown upon request.
